# A randomised pilot trial of virtual reality-based relaxation for enhancement of perioperative well-being, mood and quality of life

**DOI:** 10.1038/s41598-022-16270-8

**Published:** 2022-07-14

**Authors:** Matthias C. Schrempf, Julian Petzold, Morten Aa. Petersen, Tim Tobias Arndt, Stefan Schiele, Hugo Vachon, Dmytro Vlasenko, Sebastian Wolf, Matthias Anthuber, Gernot Müller, Florian Sommer

**Affiliations:** 1 Department of General, Visceral and Transplantation Surgery, University Hospital Augsburg, Stenglinstrasse 2, 86156 Augsburg, Germany; 2 Palliative Care Research Unit, Department of Palliative Medicine GP, Bispebjerg and Frederiksberg Hospital, University of Copenhagen, Copenhagen, Denmark; 3 Department of Computational Statistics and Data Analysis, Institute of Mathematics, University of Augsburg, Augsburg, Germany; 4 Quality of Life Department, European Organisation for Research and Treatment of Cancer, Brussels, Belgium

**Keywords:** Surgical oncology, Rehabilitation, Surgery

## Abstract

A cancer diagnosis and subsequent treatment can trigger distress, negatively impact coping resources, and affect well-being as well as quality of life. The aim of this pilot study was to investigate feasibility and clinical effects of a VR intervention on quality of life, well-being and mood in cancer patients undergoing surgery compared to a non-VR intervention and a control group. 54 patients with colorectal cancer or liver metastases from colorectal cancer undergoing elective curatively intended surgery were recruited and randomised to one of two intervention groups or a control group receiving standard treatment. Participants assigned to one of the intervention groups either received a VR-based intervention twice daily or listened to music twice daily. Adherence to the intervention was 64.6% in the music group and 81.6% in the VR group. The VR intervention significantly reduced heart rate (− 1.2 bpm; 95% CI − 2.24 to − 0.22; *p* = 0.02) and respiratory rate (− 0.7 brpm; 95% CI − 1.08 to − 0.25; *p* = 0.01). Self-reported overall mood improved in both groups (VR: + 0.79 pts; 95% CI 0.37–1.21; *p* = 0.001; music: + 0.59 pts; 95% CI 0.22–0.97; *p* = 0.004). There was no difference in quality of life between the three groups. Both interventions groups reported changes in feelings. Adherence rates favoured the VR intervention over the music group. Observed clinical outcomes showed stronger intragroup effects on mood, feelings, and vital signs in the VR group. The study demonstrated feasibility of a VR intervention in cancer patients undergoing surgery and should encourage further research investigating the potential of VR interventions to positively influence well-being and mood in cancer patients.

## Introduction

A large proportion of patients undergoing surgery for newly diagnosed cancer suffer from distress which negatively impacts quality of life^1,2^. Distress in cancer patients has been defined as a “multifactorial unpleasant experience of a psychological, social, spiritual and/or physical nature that may interfere with the ability to cope effectively with cancer, its physical symptoms, and its treatment”^3^. The prevalence of distress in cancer patients ranges from 25 to 50%, and in the general surgery population about one third of patients suffer from anxiety and half from depression^1,3–5^. There is growing evidence that distress, anxiety, and depression can adversely affect surgical and oncological outcomes and that the negative impact of untreated preoperative stress on quality of life can be measurable for up to two years after surgery^6–10^.

Colon cancer is one of the most common malignant diseases worldwide^11^. Although surgery provides good chances of cure, it has a negative impact on the patient's health-related quality of life (HRQoL) in the short and long term^12–14^. While studies in surgical oncology have traditionally focused primarily on improving oncological and surgical outcomes, today health-related quality of life (HRQoL) is considered an important outcome, especially after colorectal cancer surgery^15^. The reference values of the European Organisation for Research and Treatment of Cancer (EORTC) Quality of Life Questionnaire (QLQ-C30) further illustrate the impairments experienced by colorectal cancer patients^16,17^. Based on a large international sample of colorectal cancer patients (N = 1773), the EORTC reference values serve as a benchmark to estimate the expected scores of cancer patients in the EORTC QLQ-C30. According to these reference values, patients with colorectal cancer typically report a high burden in the HRQoL subdomains “Emotional Functioning”, “Fatigue” and “Sleeplessness”^16^.

Various interventions addressing perioperative distress, anxiety and depression have been studied. These include educational, audio-visual or relaxation interventions as well as breathing exercises, hypnosis, and cognitive behavioural therapy^18–21^. Studies suggest that perioperative stress reduction has a positive impact on quality of life and possibly positive immunological effects^22^. For instance, these immunological effects manifest themselves, in improved wound healing^22,23^.

Technological progress and advances in the development of digital therapeutics, which are interventions based on advanced technology and software programmes, have broadened the range of potential tools for stress reduction^24,25^. Virtual Reality (VR), which is increasingly being explored as a cost-effective digital therapeutic, is a technology that simulates a real environment. In a virtual environment, visual, auditory, and haptic stimuli are used to create immersion, an interactive experience, and a sense of presence in that environment^26–29^. Both realistic graphics and acoustics as well emotional responses evoked by the VR experience, contribute to the sense of presence described as a feeling of being in the virtual scene^30,31^. VR-based interventions can improve symptoms of anxiety and depression and have been successfully used to treat acute and periinterventional pain in adults and children^27,32–34^. It has also been applied to improve psychological well-being in young cancer patients^35^. Virtual immersive environments create a strong distraction as users cannot see immediate surroundings through the VR headset. Software that enables users to interact with the VR environment e.g., by steering or controlling elements of the virtual environment by head movements, can increase the degree of immersion and disctraction^28,34,36^. There is growing evidence that VR interventions can contribute to emotional, psychological, and social well-being^30^. Mindfulness-based interventions have shown to elicit positive emotional responses and improve mood^37–39^. The state of immersion that occurs in a VR environment is associated with focused attention which is intended during mindfulness practice and might enhance the effect^37^. These findings suggest that VR interventions may hold the potential to reduce perioperative stress and improve various aspects of quality of life, mood and patient satisfaction in cancer patients undergoing surgery.

Currently, little is known about the feasibility and impact of VR-based mindfulness interventions on mood, well-being and quality of life in cancer patients undergoing surgery. Therefore, the aim of this randomised pilot trial was to assess the feasibility of a VR-based intervention and to obtain estimates of the effect on quality of life, emotional and psychological well-being, patient satisfaction and surgical outcomes.

## Methods

**Figure 1:**
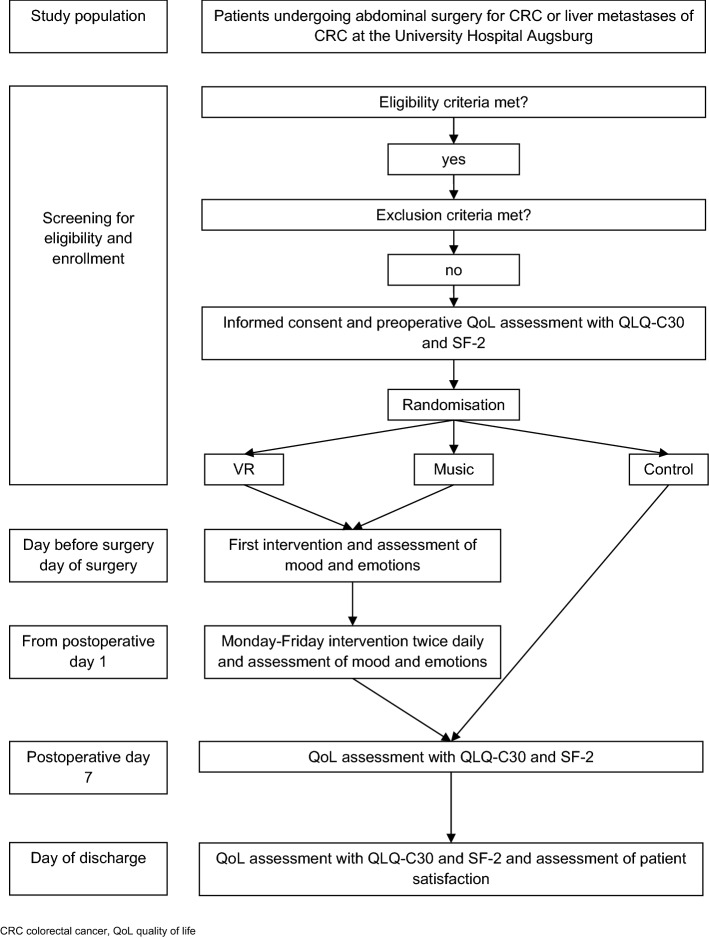
Trial flow chart.

This pilot trial was a single-centre, randomised, controlled, assessor-blinded trial featuring a 3-arm design. The trial was conducted at the Department of General, Visceral and Transplantation Surgery at the University Hospital Augsburg, Germany. The study protocol was in accordance with the Declaration of Helsinki and was approved by the Ethics Committee of the Ludwig Maximilian University Munich, Germany (reference number 19-915). The trial was prospectively registered in a primary registry in the WHO registry network (German Clinical Trials Register, registration number DRKS00020909), on 02/03/2020. The full WHO trial registration dataset is available through the WHO ICTRP search portal. The final protocol was published with open access under a Creative Commons license^40^. The introduction and methods sections are based on the published protocol^40^. The flow chart of the trial is visualized in Fig. 1.

### Participants

Written informed consent was obtained from all participants prior to participation. Patients scheduled for elective curatively intended resection of colorectal cancer (stage UICC I–III) or liver metastases from colorectal cancer (UICC IVA) aged between 18 and 75 years were eligible for participation. Exclusion criteria were inability to provide informed consent, pregnancy, emergency surgery, participation in another clinical trial with potential impact on the endpoints of this study, medical history of dementia, schizophrenia, hallucinations, panic attacks and epileptic seizures, pacemaker or defibrillator devices, intake of neuroleptic or antiepileptic drugs, active alcohol or substance abuse and lack of German or English language skills.

### Randomisation and blinding

Participants were randomised by block randomisation with the aim to reduce a selection bias and to ensure equal group sizes. A block size of 9 was chosen and not made public prior to the end of the study to prevent prediction of group allocation. The randomisation was carried out by picking a sealed opaque envelope and was performed by staff who was not involved in the data collection, data analysis or supervision of the study patients. Blinding of patients and staff supervising the study interventions did not take place due to the nature of the interventions. However, the physicians involved in the surgical and medical treatment of the patients were blinded to the interventions.

### Interventions

#### VR group

For the intervention in the VR group, a commercially available standalone VR headset (Oculus Go for Business, Facebook Technologies LLC) with external headphones and commercially available software TRIPP developed by TRIPP Inc. (TRIPP Inc., Los Angeles, USA, www.tripp.com) was used. The headset featured a 5.5″ fast-switching LCD at a 2560 × 1440 resolution (1280 × 1440 per eye). Two different VR experiences were presented to each patient in the VR group every day from Monday to Friday. Two sessions per day (morning and evening) were held. The exercises began for patients who were admitted before their surgery on the day of admission. Patients admitted on the day of surgery only had a preoperative session if the patient underwent surgery in the afternoon of the admission day. The last preoperative session was on the evening before the operation for all patients undergoing surgery in the morning, and in the morning on the day of the operation for all patients who underwent surgery in the afternoon. The first postoperative session took place on the first postoperative day or as soon as the patient was transferred from the intensive care unit (ICU) to a regular ward. The sessions were held until discharge from the hospital. During the intervention patients sat in a chair or in a hospital bed depending on patient condition and preference. The immersive VR mindfulness experiences were meditative and designed to produce a calming effect. The VR environment was updated daily to encourage ongoing engagement. Despite the daily changing environment, the morning and evening sessions consisted of recurring components that were part of every session. The morning VR experience which lasted 7–8 min, had 4 levels incorporating binaural audio, procedurally generated music, guided reflections, breathing exercises with breath visualization and a mini-game, all designed to focus attention. The audio was delivered through headphones worn by the patient. The patient was guided through the experience by a narrator. The evening VR sessions were designed to produce a more calming effect and consisted of three levels. The guided meditation content was more expansive than in the morning session. Two out of the three levels incorporated interactive breathing exercises with breath visualization. The evening sessions lasted 10 min but could be adjusted to last longer if the patient desired. Before and after the intervention, heart rate and blood pressure were measured.

#### Music group

In the music group, each participant listened to soothing classical music via a headphone from Monday to Friday twice daily for 7–10 min. Patients wore a VR-headsets which was switched off. The exercises began in patients who were admitted before their surgery on the day of admission. Patients admitted on the day of surgery only had a preoperative session if the patient underwent surgery in the afternoon of the admission day. The last preoperative session was held on the evening before the operation for all patients undergoing surgery in the morning, and in the morning on the day of the operation for all patients who underwent surgery in the afternoon. The first postoperative session took place on the first postoperative day or as soon as the patient was transferred from ICU to a regular ward. The sessions continued until discharge. Before and after the intervention, heart rate and blood pressure were measured.

#### Control group

There was no intervention in the control group.

### Outcome measures

#### Feasibility outcomes

As this study was designed and planned as a pilot trial, feasibility outcomes and clinical outcomes were assessed. Feasibility outcomes included participation, recruitment, and retention rates, questionnaire compliance, and frequency and type of adverse events.

#### Clinical outcomes

Clinical outcomes included differences in mood and feelings, as well as vital signs including heart rate, respiratory rate and blood pressure measured before and after each intervention, changes in quality of life during hospital stay and differences in quality of life between the three study groups. In addition, number and severity of surgical complications according the Clavien-Dindo classifaction and *Comprehensive Complication Index*® (CCI)^41,42^, length of hospital stay, and patient satisfaction were compared between the three study groups.

Overall mood and feelings were assessed immediately before and after each intervention using a questionnaire consisting of two questions on current feelings and overall mood. The questionnaire has been previously published and can be accessed via https://bmjopen.bmj.com/content/bmjopen/11/4/e044193.full.pdf?with-ds=yes^40^.

HRQoL was assessed in this study using the EORTC QLQ-C30^17^, a valid and widely used questionnaire in cancer patients. To improve measurement precision in the domains “emotional functioning”, “sleeplessness” and “fatigue” we included additional items selected from the EORTC CAT Core item banks (a collection of item databases) to form so-called short forms^43^. Short forms refer to a fixed short questionnaire whose items have been drawn from a calibrated item bank^44^. Items are optimally selected to build a questionnaire tailored to the population under investigation and its expected distribution on the continuum of the latent trait of interest, thus improving measurement precision^45^. Ten additional items were included to measure “sleeplessness” (3 items) “fatigue” (3 items) and “emotional functioning” (4 items) with increased precision, following recommendations of the EORTC. The ten additional items are summarised in the EORTC-SF2 questionnaire. HRQoL was assessed preoperatively before the first intervention, on postoperative day 7 and 14, and at discharge using the QLQ-C30 and EORTC-SF2. At discharge, all patients received additional questions regarding patient satisfaction. The QLQ-C30 questionnaire was scored according to the EORTC QLQ-C30 scoring manual resulting in five functional scales and nine symptom scales with scores ranging from 0 to 100. A higher score reflects better functioning/worse symptoms. The short forms for emotional functioning, sleeplessness, and fatigue were scored using the EORTC short form scoring software^43,46^ on a so-called T-score metric. This means that the European general population will have a mean of 50 and a standard deviation of 10 for the short forms and hence a score > 50 reflects better emotional functioning and worse sleeplessness/fatigue that the average general population.

### Sample size estimation

A formal sample size calculation was not performed as there is no data that could serve as a foundation for a formal sample size calculation. For pilot studies with an expected medium standardised effect size, a case number of n = 15 per group has been recommended for pilot studies preceding a possible main study with a power of 90%^47^. Therefore, 15 patients plus an additional three patients (20%) per group were enrolled to compensate for incomplete questionnaires and patients who cancel their participation in the study early.

### Statistical analysis

Continuous data is presented as mean ± standard deviation or median with interquartile range, depending on distribution. Categorical data is presented as numbers with percentages. Approximately normally distributed continuous variables were compared using the independent *t*-test. Non-normally distributed continuous variables were compared using the Mann–Whitney *U*-test. Categorical data was compared using the χ^2^ test.

For a comparison of non-normally distributed continuous variables before and after the intervention the Wilcoxon signed-rank test was used for each intervention separately. Binary data was compared before and after the intervention using McNemars test.

Analysis of clinical outcomes were performed with averaged data per participant to control for the difference in number of measurements resulting from a different length in hospital stay in addition to the analysis of unaveraged data.

Comparisons of continuous variables between three groups were performed with analysis of variance (ANOVA) or Kruskal–Wallis tests depending on distribution.

A two-sided *p* < 0.05 was considered significant. Statistical analyses were undertaken using SPSS® for Windows®, version 28 (IBM, Armonk, New York, USA) and *R*, version 4.1.1 (R Foundation for Statistical Computing, Vienna, Austria).

### Ethical approval

The study has been approved by the Ethics Committee of the Ludwig-Maximilians-University, Munich, Germany (Reference Number: 19-915). The trial was prospectively registered in a primary registry in the WHO registry network (German Clinical Trials Register, registration number DRKS00020909), on 02/03/2020. The full WHO trial registration dataset is available through the WHO ICTRP search portal (https://trialsearch.who.int/Trial2.aspx?TrialID=DRKS00020909).

### Informed consent

Written informed consent has been obtained from all participants prior to participation.

### Patient and public involvement statement

No patient involved.

## Results

### Feasibility outcomes

Between May 2020 and March 2021, a total of 54 patients were recruited and assigned to one of the three study groups. During this period 146 patients who were scheduled for elective colorectal cancer surgery were screened for study participation. 55 patients did meet not the predefined inclusion criteria and 25 patients refused study participation. Twelve patients who met the inclusion criteria were not offered participation in the study, mainly due to exceptional circumstances caused by the COVID 19 pandemic, which led to a redistribution of medical staff and changes in hygiene regulations during the study period.

The mean age of patients who refused to participate was 63.0 years compared to a mean age of 60.1 years among participants (*p* = 0.122). The proportion of women and men among participants and patients who refused participation did not differ (*p* = 0.925).

During the study period the 54 participants received 184 quality of life questionnaires, of which 175 were completed (95.1% completion rate), 94.6% of the questionnaires were completed on the scheduled day, the remaining 5.4% were completed late.

A total of 463 music interventions and 301 VR interventions were planned according to the study protocol. This difference in number of interventions was due to the longer median length of stay in the music group. 67 music interventions and 45 VR intervention were not offered due to medical or organisational reasons. Intervention adherence was significantly lower in the music group compared to the VR group (*p* < 0.001). Of the sessions offered, 256 of 396 music interventions (64.6%) and 209 of 256 VR interventions (81.6%) were conducted as scheduled. The main reason for interventions that were offered but not carried out was patient refusal, which occurred significantly more often in the music group than in the VR group (35.4% vs. 18.4%, *p* < 0.001).

Of the 209 VR sessions, one session was terminated prematurely due to nausea and vomiting, and four sessions were terminated because of technical problems. One of the 256 music interventions was discontinued prematurely because the patient had a strong emotional reaction and burst into tears.

### Demographic, medical and surgical data

Length of hospital stay, cancer stage, rate of severe complications (Clavien-Dindo ≥ III), ECOG performance status at discharge and mortality did not differ between groups. Demographic characteristics, oncological data and surgical outcomes are shown in Table 1. The intake of medication with a potential effect on outcomes including ß-blockers (*p* = 0.35), non-ß-blocker antihypertensive drugs (*p* = 0.08) and antidepressants (*p* = 1.00) did not differ between the three groups.

**Table 1:** Demographic characteristics and surgical outcomes.

	Control (n = 18)	Music (n = 18)	VR (n = 18)	*P*
**Sex, n (%)**				
Female	9 (50.0%)	4 (22.2%)	8 (44.4%)	0.3
Male	9 (50.0%)	14 (77.8%)	10 (55.6%)	
Age, mean ± SD, years	60.9 ± 10.5	63.4 ± 8.2	55.8 ± 10.2	0.04
BMI, mean ± SD, kg/m^2^	27.1 ± 6.9	28.16 ± 5.2	27.3 ± 2.9	0.46
**ASA, n (%)**				
I	9 (50.0%)	2 (11.1%)	1 (5.6%)	0.47
II	9 (50.0%)	9 (50.0%)	12 (66.7%)	
III	0	7 (38.9%)	5 (27.8%)	
**ECOG at admission, n (%)**				
0	11 (61.159	12 (66.7%)	11 (61.1%)	0.89
1	6 (33.3%)	6 (33.3%)	7 (38.9%)	
2	1 (5.6%)	0	0	
**Type of surgery, n (%)**				
Colon	8 (55.5%)	7 (38.9%)	9 (50%)	0.95
Rectum	8 (44.4%)	8 (44.4%)	8 (44.4%)	
Liver	2 (11.1%)	3 (16.7%)	1 (5.6%)	
Ostomy formation, n (%)	5 (27.8%)	8 (44.4%)	6 (33.3%)	0.68
Operating time, mean ± SD, min	176 ± 111	176 ± 60	174 ± 53	0.7
Length of hospital stay, median (IQR), days	8 (5.0–11.8)	13 (6.5–27.0)	8.5 (5.8–14.3)	0.28
**UICC stage, n (%)**				
0 or I	8 (44.4%)	8 (44.4%)	9 (50.0%)	0.81
II	3 (16.7%)	3 (16.7%)	4 (22.2%)	
III	5 (27.8%)	3 (16.7%)	4 (22.2%)	
IV	2 (11.1%)	4 (22.2%)	1 (5.6%)	
CCI®, mean ± SD	12.1 ± 21.3	28.9 ± 28.0	17.3 ± 15.8	0.049
Complication Calvien Dindo III or higher, n (%)	2 (11.1%)	7 (38.9%)	3 (16.7%)	0.18
In-hospital mortality, n (%)	0	1 (5.6%)	0	1
**ECOG at discharge, n (%)**				
0	2 (11.1%)	0	3 (16.7%)	0.16
1	10 (55.6%)	10 (55.6%)	11 (61.1%)	
2	4 (22.2%)	5 (27.8%)	3 (16.7%)	
3	2 (11.1%)	2 (11.1%)	1 (5.6%)	
4	0	0	0	
5	0	1 (5.6%)	0	

Data are mean ± SD or n (%) or median (IQR).

IQR, interquartile range; SD, standard deviation; BMI, body mass index; ASA, American Society of Anaesthesiologists; ECOG, Eastern Co-operative Oncology Group; CCI®, Comprehensive Complication Index; UICC, Union for International Cancer Control.

### Clinical outcomes

**Figure 2:**
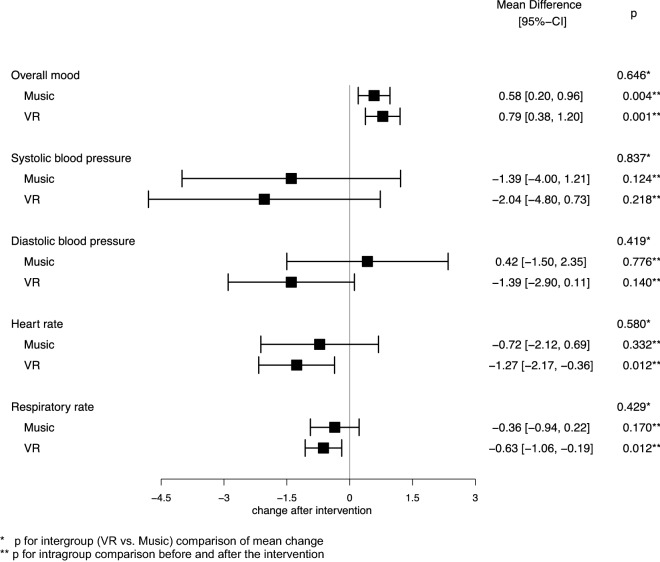
Averaged change in overall mood and vital signs after the intervention.

**Figure 3:**
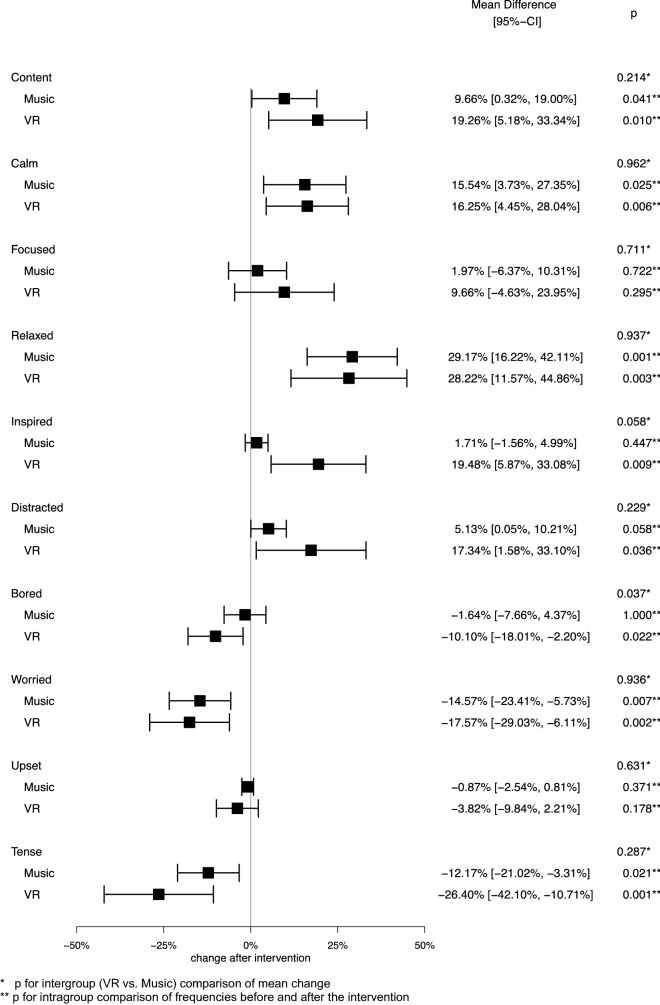
Averaged change in frequency of feelings after the intervention.

There were no differences in baseline measurements of vital signs (systolic blood pressure *p* = 0.84; diastolic blood pressure *p* = 1.0; heart rate *p* = 0.09; respiratory rate *p* = 0.77) and overall mood (*p* = 0.78) before the first intervention between the VR and music group. Analysing the averaged data per participant, heart rate and respiratory rate were significantly lower in the VR group after the intervention compared to before: − 1.3 beats per minute (bpm) (95% CI − 2.2 to − 0.4; *p* = 0.012) and − 0.6 breaths per minute (brpm) (95% CI − 1.1 to − 0.2; *p* = 0.012), respectively, while there was no difference in the music group (Fig. 2). The differences between the two groups were not significant. No significant reduction in systolic or diastolic blood pressure was seen in either group, although the trend towards blood pressure reduction was stronger in the VR group. Self-reported overall mood on a scale from 1 to 10 (higher scores reflect better mood) improved after the intervention in both groups (VR: + 0.79 pts; 95% CI 0.38–1.20; *p* = 0.001; music: + 0.58 pts; 95% CI 0.20–0.96; *p* = 0.004) but there was no difference in improvement between the two groups (*p* = 0.65). The mean difference in the frequency of feelings per participant before and after the intervention is shown in Fig. 3. Regarding intragroup comparisons, participants in the VR group felt more content (+ 19.3%; *p* = 0.010), calm (+ 16.3%; *p* = 0.006), relaxed (+ 28.2%; *p* = 0.003), inspired (+ 19.5%; *p* = 0.009), and distracted (+ 17.3%; *p* = 0.036) and felt significantly less bored (− 10.1%; *p* = 0.022), worried (− 17.6%; *p* = 0.002), and tense (− 26.4%; *p* = 0.001), after the intervention. Patients in the music group were more often content (+ 9.7%; *p* = 0.041), calm (+ 16%; *p* = 0.025) and relaxed (+ 29.2%; *p* = 0.001) as well as less worried (− 14.3%; *p* = 0.007) and tense (− 12.2%; *p* = 0.021). The corresponding 95% confidence intervals are displayed in Fig. 3. The comparison between the VR and music groups showed that participants in the VR group were significantly less bored after the intervention (*p* = 0.037), while there were no differences in other feelings.

Analysis of unaveraged data compared the effects on vital signs, overall mood and feelings between all sessions in the VR and the music groups (Fig. S1, supplemental material). In the VR group, heart rate (− 1.2 bpm; 95% CI − 2.1 to − 0.3; *p* = 0.001) and respiratory rate (− 0.7 brpm; 95% CI − 1.1 to − 0.3; *p* < 0.001) were significantly lower after the intervention. In the music group, systolic blood pressure and heart rate were significantly lower after the intervention. Compared to the VR group, the music intervention resulted in a greater reduction in systolic blood pressure when analysing the unaveraged data (− 0.6 mmHg vs. − 2.1 mmHg; *p* = 0.047). The music (+ 0.39 pts; 95% CI 0.26–0.51; *p* < 0.001) and VR intervention (+ 0.65 pts; 95% CI 0.50–0.80; *p* < 0.001) resulted in an improvement of self-reported overall mood, but the effect was significantly stronger in the VR group compared to the music group (+ 0.65 pts vs. + 0.39 pts; *p* = 0.007). In addition to the averaged data (Fig. 3), the effects on feelings were compared between the VR and music group for all sessions conducted (Fig. S2, supplemental material).

### Quality of life outcomes and patient satisfaction

**Figure 4:**
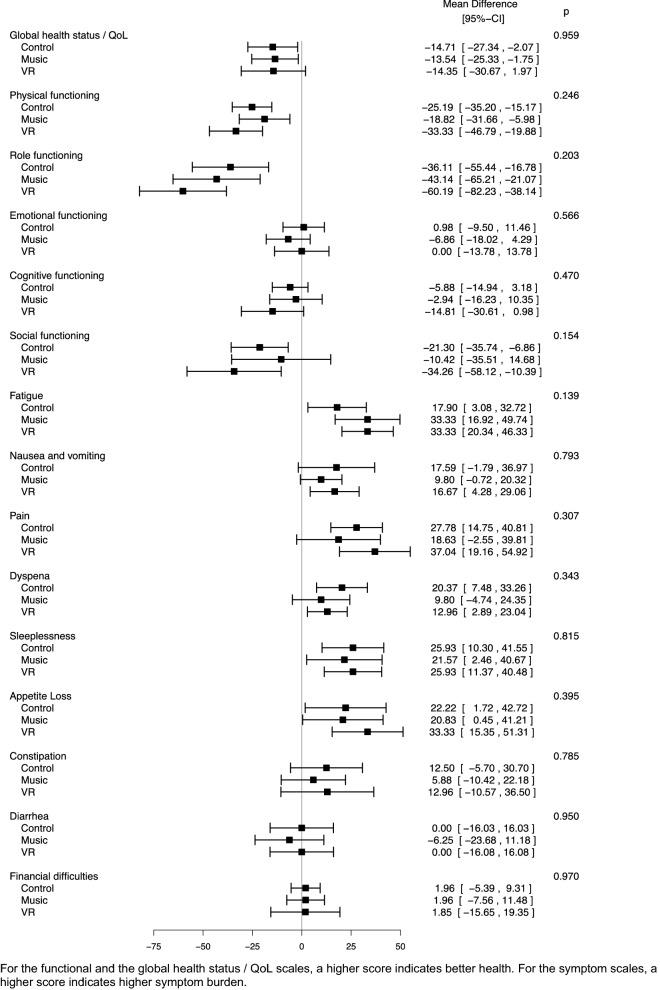
Change in quality of life between admission and discharge (EORTC QLQ-C30).

Quality of life data were evaluated for the control, music, and VR group. There were no differences in the global health and quality of life status at the time of enrolment between the three groups (*p* = 0.76). When comparing the change in quality of life between admission and discharge from hospital, there were no differences between the three groups for any of the QLQ-C30 scales (Fig. 4). The analysis of the calculated T-scores obtained after adding items to the functional scale “emotional functioning” and the two symptom scales “sleeplessness” and “fatigue” did not reveal any significant differences between the scores at admission and discharge either (Fig. S3, supplemental material). There were no differences regarding patient satisfaction at discharge between the three groups (*p* = 0.88).

## Discussion

This pilot trial delivered feasibility and clinical data on a VR intervention in a clinical setting that is typically characterized by increased levels of distress and reduced quality of life. There is growing evidence that distress, anxiety, and depression can adversely affect oncological outcomes and the negative impact of untreated perioperative distress on quality of life can be measurable for up to two years after surgery^6–10^.

In this pilot trial feasibility outcomes favoured the VR intervention over the music intervention for several reasons. Participants in the VR group were much more likely to participate in planned sessions and the main reason for not conducted music interventions was refusal of the participant which shows a higher compliance with the VR intervention.

One might argue that the music intervention could have had higher compliance rates if patients had been allowed to choose their preferred music but on the other hand participants in the VR group were not given any choice regarding the type of VR experience either.

Apart from one VR session which was stopped prematurely because of nausea no symptoms and side-effects of the VR intervention occurred during the trial. Since nausea and vomiting is a common problem after colorectal surgery, it was not possible to determine, whether the observed symptom was caused by the VR intervention or was a result of the postoperative course^48^.

In terms of the cost of the intervention, the price of the headset used in this study was $599, while the price of the consumer version of the same headset was $249 at the time of the study. A one-year business license for the software used in this study currently costs about $250. Considering that the headset can be used multiple times a day for multiple patients, the cost of the VR intervention appears negligible compared to an in-person intervention.

Both interventions resulted in a small but statistically significant reduction in heart rate and an increase in overall mood measured on a scale from 1 to 10 but no difference was observed in blood pressure changes. The magnitude of the reduction in heart rate does not appear to be clinically relevant, however, the reduction in respiratory rate in the VR group may be considered an indirect measure of relaxation. Both music and virtual reality environments have shown the ability to elicit emotions in previous studies^49–51^. In this pilot trial the VR intervention had a greater effect on overall mood than the music intervention in the analysis of unaveraged data. Therefore, the VR intervention may be more effective than the music intervention in enhancing mood and well-being. It is noteworthy that for all changes in vital signs, mood and feelings that did not reach significance, there was a stronger trend towards the desired outcome in the VR group.

The use of mindfulness-based relaxation and VR games for emotion regulation and improvement of well-being and mood is supported by other studies that have successfully used such virtual experiences^30^. Mindfulness meditation in VR environments have been shown to be more effective than standard mindfulness-based stress reduction (MBSR) in reducing pain in patients suffering from chronic pain and in increasing satisfaction in patients with traumatic brain injury^30,38,52^. Singh et al. were able to demonstrate, that VR games positively impact physical reactivation and psychological well-being in patients with physical disabilities. The software used in this pilot trial differs from previous studies as it incorporates mindfulness content such as breathing exercises and guided meditations aimed at generating mindfulness, as well as game elements, with the idea of combining both elements to enhance the desired effect. Comparing the results of this study with other studies proves to be difficult, as most studies have been conducted in healthy subjects or patient populations that differ greatly from this study^30^.

Regarding the changes in individual feelings before and after the intervention, we found several differences between the music and the VR groups. Participants in the VR group were significantly more distracted after the intervention, feeling less bored and more inspired, which was not the case after the music intervention. While “being distracted” might be considered an unfavourable outcome for the general population or in the workplace, distraction in this particular study setting was not only expected, but it was our intention to create a positive distraction from concerns regarding the malignant disease and surgical outcomes. Distraction can be defined as an “active attempt to deal with a stressful situation by engaging in an alternative pleasurable activity”^53^. Unlike avoidance, positive distraction can be regarded as an engaging and adaptive coping strategy^53,54^ and could therefore positively influence the process of coping with the cancer diagnosis and treatment related problems.

The feeling of being more inspired and less bored could partly explain the higher compliance of participants in the VR group and is probably a result of the immersive experience that encourages interaction with the VR environment.

While there was no significant difference in quality of life at discharge between the three groups, it should be kept in mind that this pilot trial was not powered to detect such differences. Considering that the median length of hospital stay was less than two weeks in all three groups, the duration of the intervention might have been too short to produce a stronger effect, especially since mindfulness-based interventions usually have a longer duration^55^. Furthermore, numerous social, mental, physical, and socioeconomic factors determine the individual quality of life.

There are several limitations that should be mentioned. The clinical outcomes of this pilot trial should be treated with caution since this trial was not powered to test for superiority of the VR intervention regarding the clinical and quality of life endpoints. While measures of quality of life, patient satisfaction and surgical outcomes were performed in all three groups, mood and vital signs were not assessed in the passive control group, which received neither the VR nor the music intervention. Furthermore, this study was conducted in a very specific patient population and setting with a relatively short application of the VR intervention. It should be noted that the VR software used was not specifically designed for this scenario and it is possible that the effects may vary in different study settings, patient groups or healthy subjects. Although the study was randomised, which should reduce the risk of accidental bias, the lack of harmonisation of pain treatment regimens could be considered a limitation.

## Conclusion

This pilot trial provided feasibility results and clinical outcomes for a VR-based intervention to improve mood and well-being for colorectal cancer patients undergoing surgery. Feasibility results favoured the VR intervention over the music intervention. Although the study was not powered to detect differences between the two intervention groups, the observed clinical outcomes showed greater improvement in mood, feelings and vital signs in the VR group than in the music group. The results should encourage further research and larger trials exploring the potential of VR interventions to positively influence disease coping processes, mood and well-being in cancer patients and patients undergoing major surgery.

## Supplementary Information

Supplementary Information 1.

Supplementary Information 2.

Supplementary Information 3.

## Data Availability

A fully anonymised data set and the statistical code can be made available upon justified scientific request and after ethical approval has been granted. Depending on the extent of the data use and the planned research, either appropriate credit or co-authorship must be granted to the authors of this study. Any requests should be addressed to the corresponding author.
