# Supplementary material for: A randomised pilot trial of virtual reality-based relaxation for enhancement of perioperative well-being, mood and quality of life
Source: Sci Rep. 2022 Jul 14;12:12067. doi: 10.1038/s41598-022-16270-8 (PMC9282619; doi:10.1038/s41598-022-16270-8)
Supplement: Supplementary file 2 — Supplementary Information 2. [file 41598_2022_16270_MOESM2_ESM.docx]

* p for intergroup (VR vs. Music) comparison of mean change

** p for intragroup comparison of frequencies before and after the intervention

Fig. S2 Unaveraged change in frequency of feelings after the intervention
