# Supplementary material for: A randomised pilot trial of virtual reality-based relaxation for enhancement of perioperative well-being, mood and quality of life
Source: Sci Rep. 2022 Jul 14;12:12067. doi: 10.1038/s41598-022-16270-8 (PMC9282619; doi:10.1038/s41598-022-16270-8)
Supplement: Supplementary file 3 — Supplementary Information 3. [file 41598_2022_16270_MOESM3_ESM.docx]

For the functional scale, a higher score indicates better health. For the symptom scales, a higher score indicates higher symptom burden.

Fig. S3 Change in calculated T-scores between admission and discharge for symptom scales sleeplessness and fatigue and functional scale emotional functioning
